# Mental Hospitals: Past and Present

**Published:** 1913-10-11

**Authors:** 


### The Hospital

The Workers' Newspaper of Administrative Medicine and Institutional Life, Administration, National Insurance and Health.

No. 1424, Vol. LV. SATURDAY, OCTOBER 11, 1913. PRICE ONE PENNY.

### MENTAL HOSPITALS: PAST AND PRESENT.

Lunatic asylums, or mad-houses, have gone through many vicissitudes during the last seventy years. A century and a-half ago the insane were exhibited at Bethlem as a show, and the public were admitted on payment of one penny or two-pence per head. Within a few years of 1800 the treatment of lunatics was notable for its cruel severity, being unintelligent and reprehensible all the world over. For the introduction of enlightened principles of treatment we are indebted to William Tuke, who established the Retreat at York, and to Pinel at Bicêtre in Paris. But, despite the action of these two philanthropists, inquiry revealed the existence of serious cruelties at Bethlem so recently as 1815. It was not, however, until 1859 that England could claim the credit of the total abolition of mechanical restraints at the best administered asylums throughout the country. Previous to that time attempts had been made by the legislature to deal with the question of lunatics and their treatment. An Act regarding criminal lunatics was passed in 1840, and in 1853 the existing laws, which were very numerous, were consolidated and amended. A new Lunacy Act for Scotland followed in 1858. In 1870 a Parliamentary Committee was appointed to report on the then system of the custody of lunatics, and its report, which was favourable to the existing system, was issued in 1878. Lord Chancellor Selborne introduced a stringent Lunacy Bill in 1885, which was re-introduced by Lord Chancellor Herschell in 1886; but it was not until 1891 that the Lunacy Acts Amendment Bill became an accomplished fact. In 1904 a Royal Commission considered the existing methods of dealing with idiots and epileptics, and with imbecile, feeble-minded, or defective persons not certified under the lunacy laws, which eventuated in the passing of the Mental Deficiency Act of 1913. On the whole, it is fair to say that the treatment of mental cases of all kinds in British asylums and hospitals at the present time is enlightened and progressive in the sense that there is a steady increase in the desire to provide for these unfortunate members of the race the most favourable conditions of treatment, with a view to secure their comfort and the recovery of those cases where treatment gives hope of marked alleviation.

We have thought that it would be helpful to issue a Special Number dealing from various points of view with mental hospitals, and questions which affect them directly and indirectly. When we spent some years in an inspection of the asylums of various countries throughout the world, we were struck with the marked difference which then prevailed in the class of inmates to be found in British and foreign asylums. In several foreign countries it was only too evident that a considerable number of patients are received into mental hospitals for treatment who, to all intents and purposes, ought never to have been admitted, because their mental condition was at least equal to that of the majority of their fellows outside, and probably of those people who, for their own purposes, had abused the law by locking their friends or relatives up in these institutions. We believe that Great Britain can claim to have secured by continuous and careful administration a system which renders abuses of this kind exceedingly rare, if not impossible. Still, cases are brought to our notice from time to time which reveal the fact that the present system of admission might be improved, for under it there is a possibility that sometimes admission to asylums may be obtained by the friends for troublesome members of their families, who, in strict fairness and justice, might safely remain at liberty. The friends of patients who really desire, before all things, to benefit the mental invalid would be wise to remember that, on the whole, the registered hospitals offer to those who can afford to pay a reasonable sum, and even to suitable cases of persons in poor circumstances, the best opportunity for adequate and up-to-date treatment. It is encouraging to know that at length we have in connection with some mental establishments well equipped and thoroughly organised pathological departments. The agitation commenced and ably promoted by Mr. R. Brudenell Carter for the establishment of a mental hospital under the control of the London County Council, though unsuccessful at the time, started a movement which has now taken root and made substantial progress, so that we may look forward with confidence to the time when provision will be made whereby every new mental case shall at once be placed under conditions which will afford the maximum of guarantees that everything which medical science and knowledge can provide shall be brought to the aid of these unfortunate sufferers.

The mental hospitals and many asylums are full of interest from the fact that they have a most excellent organisation of workshops, and that they do encourage the more capable of the inmates to exhibit their ability as artificers and in various handicrafts and higher branches of artistic and other work. We should welcome contributions from asylum workers for "The Institutional Worker," which would give the outside world some knowledge of the work which is being effected in these directions. We will gladly publish blocks illustrating the productive capacity of those engaged in work of the kind indicated, if accompanied by a short and descriptive account calculated to excite the interest of the outside world in this most useful branch of mental hospital endeavour.

